# Differential Association between HERG and KCNE1 or KCNE2

**DOI:** 10.1371/journal.pone.0000933

**Published:** 2007-09-26

**Authors:** Sung Yon Um, Thomas V. McDonald

**Affiliations:** 1 Department of Medicine, Albert Einstein College of Medicine, Bronx, New York, United States of America; 2 Department of Molecular Pharmacology, Albert Einstein College of Medicine, Bronx, New York, United States of America; Duke University Medical Center, United States of America

## Abstract

The small proteins encoded by KCNE1 and KCNE2 have both been proposed as accessory subunits for the HERG channel. Here we report our investigation into the cell biology of the KCNE-HERG interaction. In a co-expression system, KCNE1 was more readily co-precipitated with co-expressed HERG than was KCNE2. When forward protein trafficking was prevented (either by Brefeldin A or engineering an ER-retention/retrieval signal onto KCNE cDNA) the intracellular abundance of KCNE2 and its association with HERG markedly increased relative to KCNE1. HERG co-localized more completely with KCNE1 than with KCNE2 in all the membrane-processing compartments of the cell (ER, Golgi and plasma membrane). By surface labeling and confocal immunofluorescence, KCNE2 appeared more abundant at the cell surface compared to KCNE1, which exhibited greater co-localization with the ER-marker calnexin. Examination of the extracellular culture media showed that a significant amount of KCNE2 was extracellular (both soluble and membrane-vesicle-associated). Taken together, these results suggest that during biogenesis of channels HERG is more likely to assemble with KCNE1 than KCNE2 due to distinctly different trafficking rates and retention in the cell rather than differences in relative affinity. The final channel subunit constitution, *in vivo*, is likely to be determined by a combination of relative cell-to-cell expression rates and differential protein processing and trafficking.

## Introduction

The five members of the KCNE gene family encode small membrane peptides that associate with voltage-gated ion channel proteins to regulate their behavior. The best understood interaction is the complex formed by the KCNE1 product (minK) and the KCNQ1 product (KvLQT1), a combination that results in the slowly-activating delayed rectifier potassium current, I_Ks_ [1], [2]. Mutations in KCNE1 and KCNE2 have been linked to the hereditary Long QT syndrome (LQTS) [3]–[6]. KCNE2 encodes the minK-related peptide (MiRP1), which has been linked to hereditary arrhythmias and pro-arrhythmic drug sensitivity [7], [8]. KCNE1 and KCNE2 share structural similarities with considerable amino acid sequence homology (25% identity, 56% similarity), a single transmembrane domain, with an extracellular N-terminus and a cytoplasmic C-terminus [9]. The KCNE2 interaction most intensively studied has been that with the HERG channel, another LQTS-linked channel that is responsible for the rapidly-activating delayed rectifier potassium channel [Abbott, 1999 #6]. Interactions between KCNE2 and other channels however, have been described [10]–[15]. In addition to the association with KCNE2, HERG has been shown capable of associating with KCNE1 [16], [17]. Functional effects of KCNE1 on KCNQ1 current are fairly well characterized and marked. Effects of KCNE1 and KCNE2 on HERG however, are subtler. There are discrepancies among investigators describing the effects of KCNE2 on HERG currents [7], [18]–[21]. Furthermore, there is controversy regarding the tissue-specific expression of KCNE2 with conflicting reports of its existence in the heart [22]–[24] as well as temporal and spatial heterogeneity of cardiac expression [13], [15], [25]–[27]. The recent report of the KCNE2 knock out mouse raises additional possibilities regarding the role of KCNE2 in tissues other than the heart and with K^+^ channels other than HERG [28].

Most investigations concerning KCNE regulation of HERG have focused on the functional electrophysiological effects or the expression patterns of subunits *in vivo*. In the present study we sought solely to investigate the cell biological differences in KCNE1 and KCNE2 protein processing and physical interactions with the HERG channel. Such differences may help explain some of the discrepancies among groups investigating HERG/KCNE interactions.

## Methods

### Cell culture and plasmids

Human embryonic kidney 293 (HEK293) cells (from American Type Culture Collection) were cultured in RPMI 1640 (Mediatech Inc.) supplemented with L-glutamine and penicillin-streptomycin (Mediatech Inc.), plus 10% fetal calf serum (Hyclone). Cultured cells were maintained in 5% CO_2_-95% humidified air at 37°C. For analysis of KCNE interaction with HERG we generated a cell line that stably expresses HERG with the c-*myc* immmunoepitope engineered onto the C-terminus previously described [16]. Transient transfections of KCNE plasmids were performed using Lipofectamine 2000 (Invitrogen) as recommended by the manufacturer. Cells were harvested at least 48 hours after transfection to ensure adequate time for expression and association of KCNEs with HERG.

Plasmids of N-terminal FLAG-tagged wild type KCNE1, KCNE1-L51H and KCNE1 with ER-retrieval/retention signal LRRRKR [29] (KCNE1-ER) have been previously described [30]. Plasmids of wild type KCNE2 were PCR amplified from the human cDNA (a gift from Dr. SAN Goldstein) to engineer EcoR1 and BamH1 restriction enzyme sites at the 5′ and 3′ ends. The PCR product was cloned into pCR®-Blunt II-TOPO® vector (Invitrogen) and then subcloned into the EcoR1-BamH1 sites of p3XFLAG-CMV-10 vector (Sigma) resulting in a cDNA with N-terminal FLAG tag identical to the KCNE1 construct. KCNE2-ER was constructed by incorporating ER retention signal (LRRRKR) at the C-terminus of FLAG-KCNE2 WT with BD In-Fusion™ Dry-Down PCR cloning system (BD Biosciences). All the plasmids constructed for this study were fully sequenced and analyzed before use.

### Immunoblot and co-immunoprecipitation

48 hours after transient transfection, cells were washed and lysed in ice-cold NDET (1% NP-40, 0.4% deoxycholate, 5mM EDTA, 25 mM Tris-HCl, pH 7.5, and 150 mM NaCl) buffer with Complete Protease Inhibitor Cocktail Tablets (Roche Applied Science, EDTA-free) for 10 minutes, and harvested with cell scrapers. Cell lysates were centrifuged at 13,000 RPM for 10 minutes at 4°C to remove nuclei and debris. Supernatants were assayed for total protein content (Micro BCA protein assay kit; Pierce) to ensure that equivalent amounts of protein per sample were subjected to SDS-PAGE analysis. Protein samples were mixed with 4× SDS-PAGE sample buffer (4% SDS (w/v), 40% glycerol, 20% β-mercaptoethanol (v/v), 0.004% bromphenol blue (w/v), and 125 mM Tris·HCl, pH 6.8), incubated for 30 minutes at room temperature or 15 minutes at 50°C, separated on 4–15% SDS-PAGE, and transferred onto nitrocellulose membrane (Bio-Rad) either by semi-dry blotting unit (FisherBiotech) or Mini Trans-Blot™ Electrophoretic Transfer Cell (Bio-Rad). Membranes were blocked with 10% nonfat dry milk in Tris-buffered saline (TBS) for 30 minutes at room temperature and incubated with either anti-FLAG antibody diluted 1∶5000 (Sigma), anti-*myc* diluted 1∶250 (Santa Cruz), or anti-tubulin diluted 1∶10,000 (Sigma) in 5% non-fat dry milk and 0.05% Tween 20 in TBS for 2 hours at room temperature or overnight at 4°C. The membranes were then washed four times for 5 minutes each with 0.05% Tween 20 in TBS and incubated in the corresponding infrared-fluorescence IRDye® 800 conjugated donkey anti-mouse, IRDye® 700DX conjugated donkey anti-rabbit or IRDye® 700DX conjugated donkey anti-goat secondary antibodies (Rockland Immunochemicals Inc.) diluted 1∶10,000 for 30 minutes at room temperature in the dark followed by washing with 0.05% Tween 20 in TBS. The membranes were then scanned to visualize the signal at 680 nm or 780 nm by the Odyssey detection system (Li-Cor Biosciences) and densitometry of the protein bands was performed using the Odyssey software.

For co-immunoprecipitation, cells were harvested as described above, and cleared cell lysates (2 mg/total protein) were incubated with 50 µl of goat or rabbit polyclonal anti-*myc* antibody (Santa Cruz) for 2 hours at room temperature. Protein-antibody complexes were precipitated with 40 µl of protein-G-agarose (Pierce) for an additional 2 hours at room temperature. Precipitated proteins were washed with NDET buffer with protease inhibitors four times and eluted with SDS-PAGE sample buffer, separated on 4–15% SDS-PAGE, and subjected to immunoblot analysis. Goat or mouse anti-*myc* (1∶250; Santa Cruz), rabbit or mouse anti-FLAG (1∶10,000; Sigma), mouse NaK ATPase (1∶10,000; Upstate) and mouse anti-tubulin (1∶10,000; Sigma) antibodies were used to detect the corresponding proteins.

### Immunofluorescence assay

We used immunofluorescence staining of permeabilized cells to detect the cellular localization patterns of HERG and co-expressed of KCNE1 and KCNE2 proteins. Cells were plated onto glass-bottomed 35-mm dishes (Mattek Corporation) 24-hour after transfection. 48 hours after transfection, the cells were fixed with 4% paraformaldehyde at room temperature for 20 minutes, and permeabilized in 0.2% triton-X in PBS at room temperature for 10 minutes. After blocking with 5% BSA with 0.2% triton-X in PBS at room temperature for 30 minutes, the cells were washed with 0.2% triton-X in phosphate-buffered saline (PBS) at room temperature for 5 minutes three times followed by incubation with rabbit anti-*myc* (Santa Cruz; 1∶200) and mouse anti-FLAG (Sigma; 1∶4000) antibodies in 5% BSA, 0.2% triton-X in PBS at 37°C for 1 hour. The cells were washed with 0.2% triton-X in PBS at room temperature for 5 minutes three times before incubated with Alexa 488 goat anti-rabbit (Molecular Probes), and Alexa 568 goat anti-mouse (Molecular Probes) secondary antibodies (1∶8000) in 5% BSA, 0.2% triton-X in PBS at 37°C for 1 hour in the dark. To compare KCNE localization in HEK293 cells, goat anti-calnexin (Santa Cruz; 1∶2000) and mouse anti-FLAG (Sigma; 1∶2000) primary antibodies were used. The cells were washed again with 0.2% triton-X in PBS at room temperature three times, mounted in Gel/Mount™ (Biømeda Corp.) with cover slips, and kept at 4°C in the dark until subjected to fluorescence microscopy examination. Fluorescence microscopic imaging was performed using either Olympus IX70 inverted microscope (for HERG-KCNE localization), or BioRad Radiance 2000 Laser Scanning Confocal Microscope (for KCNE localization and *z*-series) with a 60×1.4 N.A. oil immersion objective lens. *Z*-stacks were generated by obtaining optical sections through the samples at 0.35 µm intervals along the z axis. For the non-permeabilized IFA, the same protocol described above was used except all the used solutions were without triton-X, and the cells were incubated with primary antibodies at 14°C in order to avoid endocytosis uptake of the antibodies. Conventional, non-confocal fluorescent images were subject to deconvolution with Powerhazebuster software (Vaytek) for parafocal noise reduction. Densitometry of HERG, KCNE, and calnexin staining across the cells was performed with the ImageJ program. Due to differing levels of expression and staining gains were adjusted to avoid saturation (35% for KCNE1 permeabilized, 23.6% for KCNE2 permeabilized, 55% for KCNE1 non-permeabilized, and 16.8% for KCNE2 non-permeabilized).

### Cell surface analyses

Cell surface protein expression was determined by labeling with the membrane-impermeant biotinylation reagent, NHS-SS-biotin (Pierce Chemical Co.) [31], [32]. Cells were washed twice with PBS containing 0.1 mM CaCl_2_ and 1 mM MgCl_2_ (PBS-CaMg), and incubated on ice in NHS-SS-biotin (1.5 mg/mL) in 20mM HEPES, pH 9.0, 2 mM CaCl_2_, and 150 mM NaCl for 20 minutes at 4°C. After labeling, the cells were rinsed briefly with PBS-CaMg and incubated in 100 mM glycine in PBS-CaMg for 20 minutes on ice to quench unreacted NHS-SS-biotin. Cells were lysed in 100 µl of lysis buffer [50 mM Tris-HCl (pH 7.5), 1% Triton X-100, 1% SDS, 150 mM NaCl, 5mM EDTA] with gentle shaking on ice for 15 min. The cell lysates were then diluted by the addition of 900 µl of lysis buffer without SDS and centrifuged for 30 minutes at 13,000 rpm at 4°C. The biotinylated proteins were precipitated from the supernatant solution with 50 µl of UltraLink® Immobilized NeutrAvidin (Pierce, Inc., Rockford, IL) and overnight incubation at 4°C with gentle agitation. The beads were washed three times with lysis buffer, twice with high-salt lysis buffer (lysis buffer with 500 mM NaCl and 0.1% Triton X-100), and once with 50 mM Tris-HCl (pH 7.5). The biotinylated surface proteins were eluted from the beads with 50 µl of SDS sample loading buffer at 25°C for 30 minutes. The remaining supernatant represented the unlabeled proteins that had not yet been trafficked to the cell surface. Proteins were then subjected to SDS-PAGE and immunoblotting.

To assay for KCNE proteins that had appeared in the extracellular environment culture media was collected from transfected cells for 48 hours and subjected to serial ultracentrifugation and immunoprecipitation of the resulting supernatants followed by SDS-PAGE and immunoblotting. SW41Ti rotor and Optima LE80K Ultracentrifuge were used to harvest the membrane vesicles from the cleared medium at 100,000×g, for 2 hours at 4°C [32]. The vesicle pellets were washed in ice-cold PBS and re-centrifuged at 100,000×g for 1-hour at 4°C in order to remove residual media. After ultracentrifugation, the protease inhibitors were added to the supernatant, which was pre-cleared with 30 µl of mouse IgG agarose (Sigma) for 30 minutes at room temperature. The cleared supernatant was then incubated with 240 µl of EZview™ Red anti-FLAG M2 affinity gel (Sigma) overnight at 4°C with rocking. The gel was collected and washed with TBS containing protease inhibitors at 4°C for 5 minutes three times, and the protein was eluted from the gel by addition of 240 µl of 3XFLAG peptide (150 ng/µl) in TBS at 4°C for 30 minutes, and then by addition of 30 µl of 1.0 M glycine HCl (pH 3.5) at room temperature for 5 minutes. Supernatant containing the protein was separated by centrifugation at 8,200 g for 5 minutes and was neutralized by addition of 30 µl of 0.5 M Tris HCl, pH 7.4, 1.5 M NaCl, followed by immunoblot analysis using rabbit anti-FLAG (1∶3000, Santa Cruz) to detect FLAG-KCNE protein.

### PNGase F and Endo H digestion

KCNE proteins from the cell lysates were immunoprecipitated with rabbit anti-FLAG antibody. The precipitated protein-antibody complexes were incubated with denaturing buffer (0.5% SDS, 1% β-mercaptoethanol) at 100°C for 10 minutes. Samples were then digested by the addition of 5000 units of either PNGase F (New England Biolabs) in 50 mM sodium phosphate (pH 7.5) with 1% NP-40, or Endo H (New England Biolabs) in 50 mM sodium citrate (pH 5.5) for 2 hours at 37°C. Some of the proteins were subjected to double digestion with both endoglycosidases for 2 hours at 37°C. The digested proteins were separated on 4–15% SDS-PAGE and subjected to immunoblot analysis.

### Mass Spectroscopy analysis

The 20 kDa and 40 kDa bands of KCNE2 from the cell culture media were separated on 4–15% SDS-PAGE and visualized on gel by silver staining method (Silver Stain Plus kit, Bio-Rad). The bands were cut, destained and in-gel digested with trypsin before being subjected to liquid chromatography/tandem mass spectrometry analysis. Reverse phase HPLC separation was performed on a Nano LC System (Dionex). Digested proteins were diluted 3-fold in mobile phase solvent A (2% acetonitrile and 0.1% formic acid in H_2_O, v/v) and loaded on a PepMap C_18_ pre-column (5 µm, 100Å, 300 µm i.d.×5 mm, Dionex). The pre-column was washed with solvent A for desalting and then peptides were forward flushed into a C_18_ nano column (3 µm, 100Å, 75 µm i.d.×150 mm, Dionex). The peptides were eluted at a flow rate of 250 nL/min with a gradient from 5–55% solvent B (20% water and 0.1% formic acid in acetonitrile, v/v) over 50 minutes, 55% solvent B for 10 minutes and then to 95% solvent B in 10 min. The eluate from the HPLC column was directly fed into an API Q-Star Pulsar-i quadruple time-of-flight mass spectrometer (Applied Biosystems/MDS, Foster City, CA) equipped with a nanospray ionization source. For MS/MS experiments, precursor ions were selected automatically by the Information Dependent Acquisition method from AnalystQS® software (Applied Biosystems/MDS, Foster City, CA). The single most intense ion was selected to be fragmented from the mass spectrometry (MS) survey scan. For data analysis, peak lists were generated using Analyst QS® and searched against NCBInr database using Mascot® (Matrix Science, Boston, MA). Peptide matches obtained from this analysis were confirmed by *de novo* sequencing of the corresponding MS/MS spectra.

## Results

### KCNE1 and KCNE2 co-expression and association with HERG

**Figure 1:**
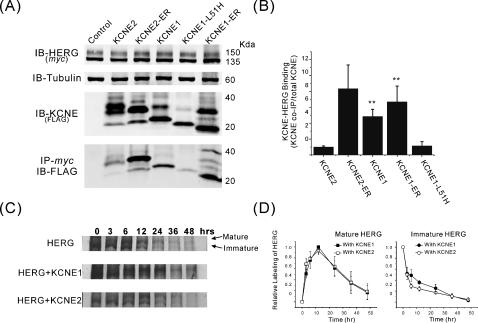
Physical association of HERG with KCNE1 and KCNE2. (A) Immunoblots of HERG-*myc* and FLAG-KCNE proteins co-transfected into HEK cells stably expressing HERG. Top gels show HERG and endogenous tubulin as a loading control. Lower gels show immunoblots of KCNE proteins from cell lysates (IB-KCNE/(FLAG)) and of KCNE that co-precipitated with HERG (IP-*myc*/IB-FLAG). (B) Densitometry analysis of efficiency of association of KCNEs with HERG expressed as amount of KCNE co-precipitated divided by the total amount of KCNE in the cells [KCNE-IP/(KCNE from cell lysates/tubulin)] (for comparison, results were normalized to 1 for KCNE2). (**: *p*<0.005, n = 3). (C) Metabolic labeling of HERG with ^35^S and pulse-chase from cells co-transfected with either GFP as control (top gel), KCNE1 (middle gel) or KCNE2 (lower gel). (D) Densitometry analysis of pulse chase (normalized to maximum label intensity within experiment). Left panel shows time course of appearance and disappearance of the higher molecular weight mature form of HERG. Right panel shows time course of disappearance of lower molecular weight immature form of HERG (n = 4).

To examine the effects of KCNE trafficking on their association with HERG, we engineered an ER retention signal at the C-terminus of the KCNE expression vectors (KCNE1-ER and KCNE2-ER) [30]. We also employed the LQT mutant KCNE1-L51H, which confers a misfolding-dependent trafficking defect [30], [33]. When transfected to HEK293 cells stably expressing HERG channel proteins (HERG-HEK), KCNE1 and KCNE2 each exhibit multiple bands on SDS-PAGE immunoblots representing various glycosylation states as previously described (Figure 1A). Expression of each trafficking mutants KCNE1-ER and KCNE2-ER was confirmed by immunoblot showing multiple glycosylation forms consistent with their altered subcellular location (Figure 1A). As previously reported for KCNE1 [30], the ER-retention/retrieval signal of KCNE2 results in increased amounts of higher molecular weight bands consistent with a protein that can progress to early Golgi compartments but is retrieved into the ER allowing prolonged exposure to glycosylation enzymes. The ER retention/retrieval signal also resulted in generally greater abundance of KCNE proteins compared to wild type (46% and 78% increase of KCNE1 and KCNE2, respectively). In contrast, KCNE1-L51H showed lower expression amounts with a primarily immature form consistent with a severe trafficking defect as previously shown [30], [33].

When HERG was immunoprecipitated and assayed for co-precipitated KCNE proteins evidence for physical association with each of the KCNE1 and KCNE2 and their mutants was detected (Figure 1A, lower panel). We quantified the amount of KCNE protein that associated with HERG by taking the densitometry value of precipitated KCNE proteins divided by total KCNE protein expressed in the cell lysates normalized to tubulin. This measure of relative binding of KCNEs to HERG (summarized in Figure 1B) showed that KCNE1 more readily associated with HERG than KCNE2. Addition of ER-retention/retrieval signal to the KCNEs resulted in greater association of the proteins with HERG. The overall degree of association of HERG with KCNE1-ER and KCNE2-ER was comparable however, the increase over wild type was greater for KCNE2 than for KCNE1. Given that the ER-retention signal increases the duration of KCNE2 in the ER these results are in agreement with results that previously showed KCNE1 association with KCNQ1 primarily occurring in the ER [30]. Moreover, the markedly increased binding of HERG with KCNE2 upon ER retention suggests that differences between KCNE1 and KCNE2 integration into the channel may be more a function of opportunity for interaction than different affinities. KCNE1-L51H displayed similar degree of binding affinity to HERG as KCNE2 wild type, but much lower than seen with KCNE1 wild type or the ER-retention/retrieval mutants. This is likely due to cellular quality control of misfolded proteins with rapid shunting the mutant protein towards degradation pathways [30], [33].

To examine potential effects of KCNE1 and KCNE2 association on HERG posttranslational processing, ^35^S-labeling and pulse-chase were performed (Figure 1D). The overall turnover rate of HERG is approximately 12 hours which neither KCNE1 nor KCNE2 co-expression altered. The conversion of the lower molecular weight species of HERG (immature glycosylation) to the more mature higher molecular weight form however appeared slightly delayed when KCNE1 was co-expressed compared to KCNE2. Since the lower molecular weight species of HERG has been proposed to represent a fraction that is immature and still being trafficked through the ER/Golgi system [34], its persistence during the pulse-chase may reflect co-assembly and reduced forward trafficking rate conferred onto HERG by KCNE1.

### Subcellular localization of KCNE1, KCNE2 and HERG

**Figure 2:**
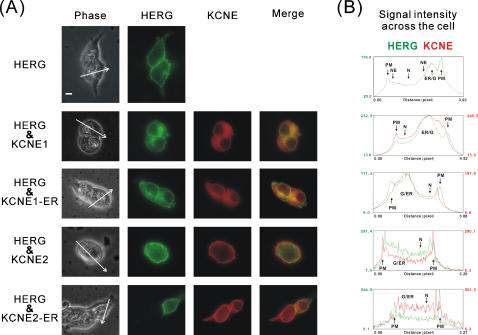
Subcellular distribution of KCNE1 and KCNE2 relative to HERG. (A) Immunofluorescence micrographs of HEK293 cells co-transfected with indicated KCNE proteins and HERG. The green channel shows staining for HERG-*myc*, the red channel shows staining for FLAG-KCNE; the merged photos show the combination. Scale bar, 10 μm, fluorescence micrographs were exposed for equal amount of time within each wavelength. The left side photomicrograph shows the visible light phase pictures with lines drawn across a cell to indicate the plane of analysis for B. (B) Densitometry of KCNE and HERG signals across diameter of the cell as indicated by the arrows from A-Phase pictures performed with ImageJ software. The X-axis is the scanned cellular distance of each cell in pixels, and Y-axis indicates the relative fluorescence intensity for each channel in arbitrary units. Abbreviations used are as follows: PM, plasma membrane; G, Golgi apparatus; NE, nuclear envelope; N, nucleus; ER, endoplasmic reticulum.

Since ER-retention/retrieval altered KCNE binding to HERG we next examined localization of the various KCNE subunits with respect to HERG in double-staining immunofluorescence analysis. HERG, in the absence of KCNEs, was localized on the cell surface but with a significant fraction consistently appearing in intracellular locales corresponding to ER and Golgi compartments representing HERG at various stages of maturation and trafficking. Double-staining of KCNEs and HERG showed a different pattern of co-localization for KCNE1 and KCNE2 (Figure 2A). KCNE1 had considerable overlap with HERG in all subcellular locales including on the surface. KCNE2, however, showed very little staining in intracellular compartments but prominent staining at the very periphery of the cell. Close examination (Figure 2B) of the staining patterns of KCNE and HERG revealed that KCNE2 showed some overlap with surface HERG but also seemed to exist at the edges of the cell independently from HERG. KCNE1-ER showed little or no co-localization with HERG at the cell surface but prominent overlap in the intracellular compartments. KCNE2-ER co-localization pattern was nearly the converse of wild type KCNE2 with prominent intracellular staining pattern and no overlap with HERG at the cell surface.

**Figure 3:**
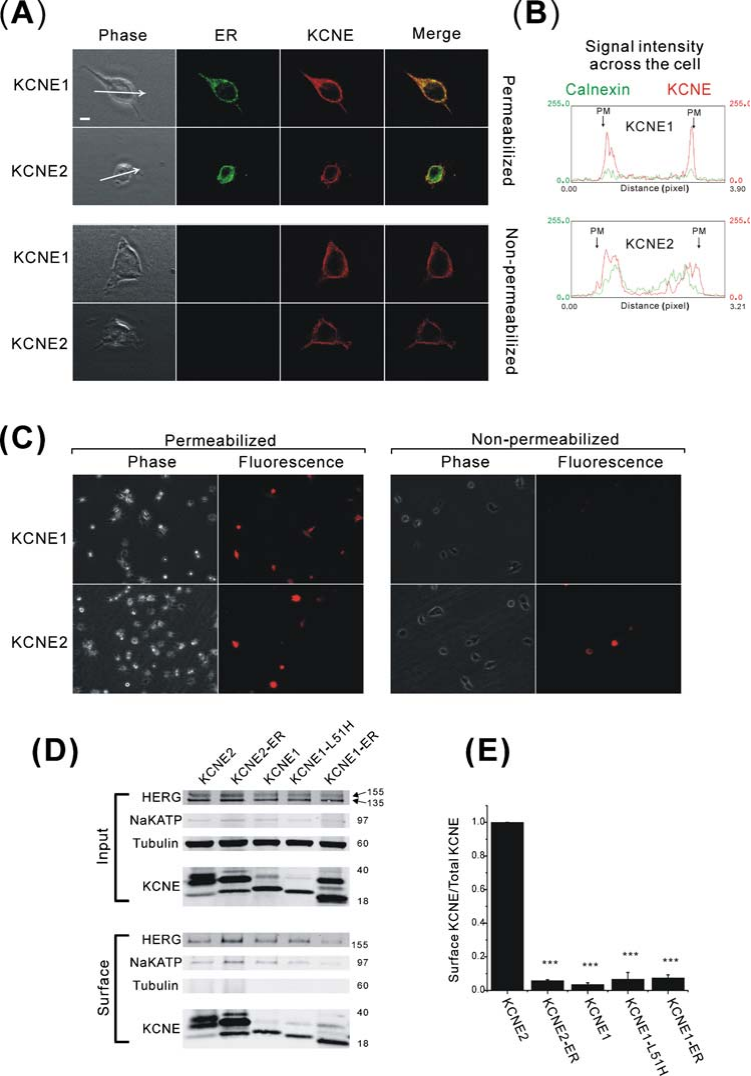
Cell surface presentation of KCNE proteins. (A) Confocal immunofluorescence micrographs of HEK293 cells transfected with ether KCNE1 or KCNE2 and counter-stained with anti-calnexin antibody to indicate the ER. Upper 2 rows show cells stained after permeabilization (KCNE1 micrographs exposed at 1.5× higher gain than KCNE2). Lower 2 rows show cells stained without permeabilization. (KCNE1 micrographs exposed at 3.3× higher gain than KCNE2). Animation of 3-D reconstruction of KCNE distribution relative to ER staining may be seen in supporting animation files Animation S1 (KCNE1-calnexin) and Animation S2 (KCNE2-calnexin). (B) Densitometry of KCNE and calnexin (ER) signals across diameter of the cell as indicated by the arrows from panel A-Phase pictures (permeabilized) performed with ImageJ software. The X-axis is the scanned cellular distance of each cell in pixels, and Y-axis indicates the relative fluorescence intensity for each channel in arbitrary units. (C) Lower magnification of HEK-HERG cells transfected with KCNE1 or KCNE2 (left panels permeabilized and right panels non-permeabilized: micrographs exposed at the same gain and exposure time for quantitative comparison). (D) Immunoblots of total KCNE and tubulin loading control from cell lysates (top gels) and surface proteins isolated by NHS-biotin labeling and precipitation (lower gel). Tubulin serves as negative control for surface labeling and NaKATPase as positive control for surface labeling in lower panels. (E) Densitometry analysis of the efficiency of surface presentation of KCNE proteins expressed as the amount of surface KCNE divided by the total cellular KCNE normalized for loading with tubulin [surface KCNE/(input KCNE/tubulin)] (comparison across groups normalized to the value of KCNE2-WT). (*: *p*<0.005; and **. *p*<0.001).

The different co-localization of KCNE1 and KCNE2 with respect to HERG prompted us to examine their location relative a known ER-resident protein, calnexin. Confocal microscopy with double-staining showed that KCNE1 co-localized more completely with calnexin than did KCNE2 with a distinct preference of KCNE2 on the surface (Figure 3A,B). The difference was more obvious when cells were not permeabilized prior to immunostaining. KCNE2 staining was more intense than that of KCNE1 and a higher percentage of cells expressing KCNE2 showed evidence for surface staining compared to KCNE1 (100% of transfected cells positive for surface staining for KCNE2 compared to 81.3±5.7% for KCNE1, n = 5 independent experiments) (Figure 3C). In another assay for surface presentation of the KCNE proteins, intact cells were subjected to surface-labeling with biotin followed by streptavidin-precipitation prior to cell lysis and SDS-PAGE analysis. Results showed that the KCNE proteins were present on the cell surface to varying degrees (Figure 3D). To quantify the relative amounts of surface presentation densitometry analysis was performed where the amount of surface (biotinylated) KCNE protein was divided by the amount of KCNE protein from the whole cell lysates (normalized to cellular tubulin) (Figure 3E). This analysis showed that wild type KCNE2 was the most efficiently presented to the cell surface of all the subunits. Initially it was surprising to see evidence of surface labeling of KCNE1-ER and KCNE2-ER however the efficiency was fairly low compared to wild type KCNE2 and may represent some escape from the ER-retention/retrieval machinery induced by the high level of forced expression in transient transfection. Internal protein controls (tubulin) were not detected among the isolated surface proteins by immunoblotting whereas native NaKATPase was.

### Blocked ER to Golgi traffic alters KCNE-HERG association

**Figure 4:**
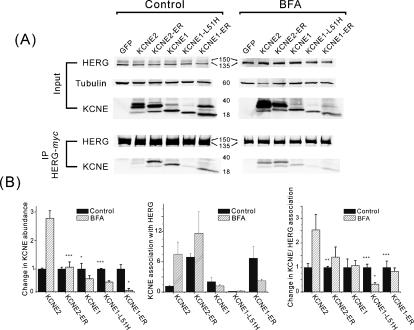
Brefeldin A trafficking block enhances KCNE2 association with HERG. (A) Immunoblots of HERG and KCNE proteins from total cell lysates (top gels, Input) and from HERG-*myc* co-immunoprecipitation (lower gels, IP HERG-*myc*). Untreated, control cells on the left and BFA-treated (36 hours) cells on the right. (B) Densitometry analyses of results. Left panel shows the relative change in KCNE abundance after BFA treatment normalized to control values. Middle panel shows the degree of KCNE association with HERG (KCNE co-IP/[total cellular KCNE/tubulin]). Right panel shows the relative change in KCNE association with HERG after BFA treatment normalized to control values. Filled columns represent untreated control values and hatched columns represent values after BFA treatment. Densities in left and middle plots are normalized to tubulin and in right plot normalized to co-immunoprecipitated HERG. (asterisks indicate significant difference from KCNE2-WT: *: *p*<0.05; **: *p*<0.005; and ***: *p*<0.001).

The combination of increased KCNE2-ER abundance and enhanced association with HERG suggested that KCNE2 was trafficked differently than KCNE1 and HERG and that, if forced to reside in an early compartment long enough, the interaction with HERG would be more likely to occur. To further investigate this possibility without KCNE mutations we employed Brefeldin A (BFA), an inhibitor of forward protein trafficking that results in collapse of Golgi stacks into the ER [35]. Nine hours after transfection with KCNE plasmids, 3 ng/ml of BFA was administered to the cells for an additional 36 hours (a concentration 3–4 orders of magnitude below those reported to cause apoptosis [36]) before harvesting for immunoprecipitation and immunoblot analyses. BFA treatment resulted in several changes in KCNE and HERG proteins (Figure 4). One of the most obvious was the altered gel mobility of the proteins, likely due to altered glycosylation. The usual 150 kDa and 135 kDa HERG species (representing mature glycosylated and immature core glycosylated forms, respectively) were replaced by a single band that was intermediate in gel mobility (Figure 4A). This is consistent with the movement of *cis*-Golgi-resident glycosylation enzymes into to the ER during BFA treatment [37]–[39]. There was also a marked increase in the abundance of wild type KCNE2 with a predominance of the higher glycosylation forms (Figure 4A, B). This effect did not occur with KCNE1 or KCNE1-ER (Figure 4B, left panel). When HERG was immunoprecipitated the amount of KCNE2 that co-precipitated was strikingly increased whereas the amount of KCNE1 that associated with HERG was not significantly altered (Figure 4B, middle and right panels). BFA treatment also resulted in enhanced KCNE2-ER binding to HERG but to a much lower degree than that seen with wild type KCNE2 (Figure 4B, right panel). These results are comparable to those obtained with ER-retention mutants.

### Appearance of extracellular KCNE2

**Figure 5:**
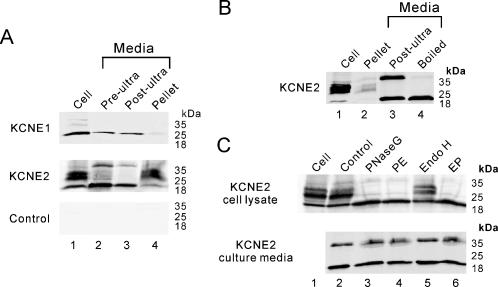
Extracellular KCNE proteins. (A) Immunoblots of KCNE proteins from the cell culture media. Gels show results from cells transfected with KCNE1 (top), KCNE2 (middle), and GFP as control (bottom). Lane 1 shows proteins from whole cell lysates. Media was subjected to FLAG-KCNE immunoprecipitation prior to immunoblot either before (pre-ultra, lane 2) or after (post-ultra, lane 3) ultracentrifugation. Lane 4 shows material from the ultracentrifuge pellet. (B) Similar analysis as in A, but for KCNE2 only, with lane showing cell lysate proteins, and lane 2 showing ultracentrifuge pellet fraction. The post-ultracentrifugation supernatant was subjected to FLAG-KCNE immunoprecipitation and loaded directly (lane 3) or after boiling for 5 minutes in sample-loading buffer (lane 4). (C) Endoglycosidases analysis of cellular (top gel) and extracellular (bottom gel) KCNE2 proteins is shown in immunoblots. Lane 1 shows cell proteins untreated; lane 2 proteins incubated in enzyme buffer without enzyme; lane 3 proteins treated with PNGase F; lane 4 proteins sequentially treated with PNGase F then Endo H (PE); lane 5 proteins treated with Endo H, and lane 6 proteins sequentially treated with Endo H then PNGase F (EP).

**Figure 6:**
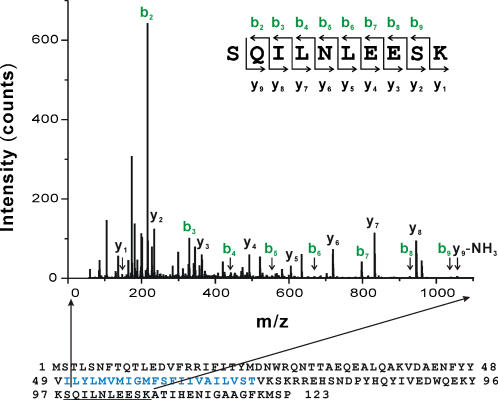
MS/MS spectrum of extracellular KCNE2 proteins. MS/MS spectrum of a tryptic peptide from the 40kDa band immunoprecipitated from KCNE2-expressing cell culture media is shown. On the top, the peptide sequence shows the location of the individual ions that are labeled in the spectrum. The bottom of the figure shows the whole sequence of KCNE2 with detected peptide underlined and transmembrane region in blue. Abbreviations used are as follows: m/z = (MW+nH^+^)/n where m/z = the mass-to-charge ratio marked on the abscissa of the spectrum; MW = the molecular mass of the sample, n = the integer number of charges on the ions, H = the mass of a proton = 1.008 Da. Ions labeled *b* in green color are fragment ions for each amino acid residue having the charge retained on the N-terminal fragment at the CO-NH bonds cleaved, and the ions labeled *y* in black color having the charge on the C-terminal fragment. The mass difference between two adjacent b ions, or y ions, is indicative of a particular amino acid residue.

Given the predominant appearance of KCNE2 at the surface of the cell surface (with a curiously more peripheral staining than HERG) and the increase in cellular KCNE2 abundance with ER-retention or blocked forward trafficking we sought to determine if the subunit was being exported out of the cell. Accordingly, we examined culture media from cells expressing KCNE subunits for appearance of KCNE proteins. To assay for both membrane-bound and free KCNE proteins we performed ultracentrifugation and immunoprecipitation from the culture media after it was gently centrifuged to clear the media of dead cells and debris. Immuno-reactivity of both KCNE1 and KCNE2 was detected in the media although KCNE2 appeared much more abundant (Figure 5A). The post-ultracentrifugation pellet contained a portion of the KCNE proteins that primarily consisted of the higher glycosylation forms compared to those seen in the cell lysates. The KCNE2 protein that was immunoprecipitated from the post-centrifugation supernatant consisted of a smaller 20 kDa species (at the same gel migration as the lowest glycosylation from cell lysates) and a 40 kDa species (not represented in the cell lysates). When samples immunoprecipitated from the media were boiled in loading buffer prior to electrophoresis, the 40 kDa band collapsed into the 20 kDa species suggesting the existence of a dimer (Figure 5B). Furthermore, the 40 kDa band did not represent a higher glycosylation form as it was resistant to Endo-H and PNGase-F digestion (Figure 5C). LC-MS results indicate that both the 20 kDa and 40 kDa bands represented the same KCNE2 fragment without significant contamination from other proteins (Figure 6). Furthermore, the KCNE2 peptide fragment identified by the MS-MS analysis is located close to the C-terminus in the cytoplasmic part of the protein (Figure 6), which argues that the lower mass protein is not a degradation product, since immunoprecipitation was via the N-terminal epitope. Immuno-reactivity for HERG was not detected in any of the culture media fractions.

## Discussion

**Figure 7:**
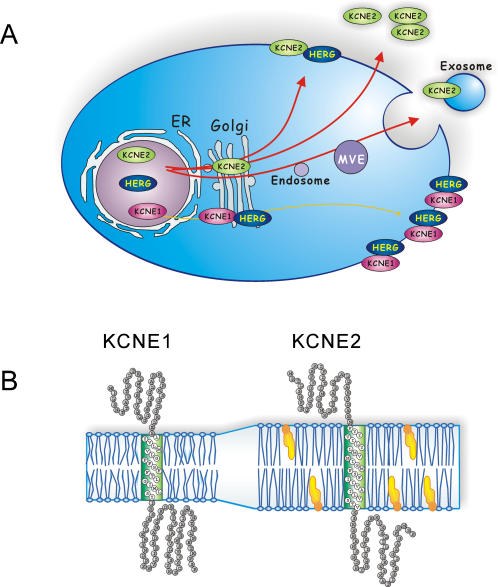
Proposed model and mechanism of KCNE1 and KCNE2 trafficking differences and association with HERG. (A) KCNE2 is trafficked onto the surface membrane at a faster speed (represented as heavy red arrows) by mechanisms including exosomes and multivesicular endosomes (MVE). KCNE1 and HERG exhibit slower trafficking rates (represented as a thin yellow arrow) with more prolonged times in the ER and Golgi thereby allowing for greater opportunity to co-assemble. Rapid transport of KCNE2 to the cell surface is accompanied by exosomes export and appearance of extracellular proteins that may exist as monomers or oligomers. (B) An illustration of possible differences in transmembrane segment length between KCNE1 and KCNE2 with more favorable energetic environment for KCNE2 in thicker cholesterol-rich plasma membrane.

KCNE1 and KCNE2 are accessory proteins that bind to various voltage-gated potassium channel pore-forming α-subunits. Their possible *in vivo* association with the HERG channel has considerable significance in terms of cardiac rhythm control, inherited arrhythmias and pro-arrhythmic drug sensitivity [9]. There are still questions and controversies regarding the functional effects of KCNE proteins on the HERG channel and much has been published on the electrophysiological effects of these associations [7], [16], [18]–[21]. Pressing questions remain concerning *in vivo* tissue expression and specificity of association [13], [15], [17], [22]–[28]. In this study we have focused on the physical protein-protein association and trafficking of KCNE1 and KCNE2 with HERG. Our results show that in the HEK heterologous expression system KCNE1 associates with HERG more robustly than KCNE2. This difference is abolished if forward trafficking of KCNE2 is slowed or blocked by molecular or chemical methods. Prevention of forward trafficking predictably altered the subcellular distribution of KCNE2 from its prominent peripheral localization towards the intracellular compartments where it acquired considerable overlap with HERG. Manipulation of forward trafficking also caused a distinct increase in cellular accumulation of KCNE2; an effect that was not seen with KCNE1 or HERG. A considerable fraction of KCNE2 also appears exported from the cell as both membrane bound and free forms. Taken together these results suggest that KCNE2 is more rapidly processed and trafficked to the cell surface than either KCNE1 or HERG (Figure 7A). The result is a reduced opportunity for KCNE2 interaction with HERG as compared to KCNE1 despite seemingly similar affinities for the channel protein.

That KCNE2 binding to HERG was markedly enhanced by prevention of its forward trafficking supports the concept that assembly of KCNE proteins with K^+^ channels happens early in the biosynthetic process, probably in the ER. It has been suggested by other groups that KCNE proteins may be capable of entering a channel complex with KCNQ1 channels preformed in the plasma membrane using the *Xenopus* oocyte expression system [40]. KCNQ1 and KCNE1 trafficking mutants in mammalian cell systems have provided evidence that assembly occurs early in the ER [30], a finding subsequently confirmed by another group [41]. Precedence for early ER assembly of other K^+^ channel subunits exists. The ER was shown as site of voltage-dependent Shaker-type K^+^ channel synthesis, tetramer assembly and processing [42]–[44]. The incorporation of KCNE1 into KCNQ1 channels with a possible stoichiometry of 2:4 KCNE1∶KCNQ1 has been suggested as a sequential process of dimerization of dimers which is also likely to occur within the ER [45], [46]. The actual site(s) of final subunit assembly however may also include early Golgi compartments since the ER-retention/retrieval mutants of KCNE1 and KCNE2 probably enter the Golgi and are repeatedly retrieved into the ER. That KCNE1-L51H showed decreased binding to HERG supports this since its behavior suggests that it never advances into any Golgi compartment but is likely shunted to the quality control mechanisms for early (possibly co-translational) degradation [47]. An alternative interpretation however, is that mis-folding of KCNE1-L51H may reduce its affinity for HERG channel proteins.

The abundance of KCNE2 on the cell surface compared to intracellular compartments was much greater than that of either KCNE1 or HERG. This suggests that its forward trafficking rate differs from the other two proteins. That it appeared on the surface at a location even more peripheral than co-expressed HERG and that a fraction appeared exported from the cell leads us to consider alternative routes of traffic and secretion. We analyzed KCNE1 and KCNE2 for possible secretory signal sequences, non-classical secretory protein prediction, (SignalP, SecretomeP, http://www.cbs.dtu.dk/services) which showed that the N-termini of KCNE1 and KCNE2 had a moderate probability of representing secretion signal sequences but no likely signal peptide cleavage sites. Another established method for export of integral membrane proteins is via exosomes, small membrane vesicles that are transported to the cell exterior via multi-vesicular endosome (MVE) pathways. MVEs, in some cases, traffic directly to the cell surface from the ER, bypassing Golgi compartments [48]. Exosomes originate from endosomes as intraluminal vesicles [49], and can carry various and unique subsets of membrane and cellular proteins [50]. Exosome secretion participates in the eradication of obsolete proteins [49] but recent findings indicate that exosomes constitute a potential mode of intercellular communication, as in the immune system [51]. Release of exosomes by tumor cells [52] and their implication in the propagation of pathogens such as retroviruses [53] and prions [54] suggests their participation in pathological situations. Recent studies show that human kidney tubules secrete exosomes containing aquaporin-2 water channels, epithelial sodium channels and other apical plasma-membrane transporters into the urine [55]. In our study, the ultracentrifugation fractions (the established method to collect exosomes) contained glycosylated KCNE2 proteins comparable to those from total cell lysates (Figure 5), supporting exosome-mediated transport of a portion of KCNE2 proteins to the extracellular environment. Since both of KCNE1 and KCNE2 were detected from exosome fractions, exosomes may not be the KCNE2-specific mechanism involved in preferential export. The abundance of KCNE2 in exosome fractions however, was greater than KCNE1 (Figure 5).

By what mechanism do KCNE1 and KCNE2 move differently through the cell? There are several possibilities. Amino acid sequence-specific sorting signals have been described within the transmembrane domains and cytoplasmic domains of the membrane proteins [56]. An alternative mechanism that does not employ specific signal sequences involves the chain length and hydrophobicity of transmembrane segments of membrane proteins. This hypothesis stems from the observations that membrane proteins exiting the Golgi destined for the cell surface have longer transmembrane domains than those staying resident in ER and *cis*-Golgi compartments [57]–[59]. In these studies transmembrane domains of plasma membrane proteins analyzed had a generally greater length (on average 21 residues) whereas the Golgi enzymes have consistently shorter membrane-spanning regions of approximately 17 residues, and are frequently enriched in residues with large side chains such as phenylalanine residues [57], [59]. Transmembrane domain length also determines membrane targeting of syntaxin 3, 4 and 5 [60]. The *cis*-Golgi/ER localization of syntaxin 5 was determined by a transmembrane segment length of 17 amino acids, whereas the surface membrane locale of syntaxins 3 and 4 were dictated by segments of 23–25 residues in length. Differences in the lipid composition and bilayer thickness of ER, Golgi and plasma membranes underlie the proposed mechanism by which transmembrane segment length determines efficiency of forward trafficking. ER and *cis*-Golgi membranes are rich in phosphoglycerides and have low amounts of cholesterol and glycosphingolipids with an estimated thickness of ∼37Å. The plasma membrane is more abundant in cholesterol, glycosphingolipids and has a higher degree of fatty acid chain saturation with an estimated membrane thickness of ∼43Å [61]. Specialized areas of the plasma membrane that are highly enriched in these constituents are lipid rafts. There is a gradient of transition of these membrane components from ER through the Golgi. The efficiency of transition through the secretory pathway of membrane proteins may be determined by the more energetically favorable milieu: fewer hydrophobic residues of long transmembrane segments will be exposed to aqueous environment in thicker lipid bilayers. KCNE1 and KCNE2 do not have obvious retention signals within their transmembrane domains or C-termini by database searches to date. Prediction of transmembrane helices of KCNE1 and KCNE2 from amino acid sequence by analysis programs suggests that the transmembrane domain of KCNE2 may be 3-4 residues longer than that of KCNE1 (25 for KCNE2 and 21 for KCNE1, Table 1 and Figure 7B). Although at 21 residues the transmembrane helix of KCNE1 should allow for transit to the surface, it may move through the Golgi less efficiently than KCNE2, thereby explaining our findings. Clearly, further investigation is warranted to determine if differential trafficking of KCNE1 and KCNE2 are controlled by signal sequences versus sequence-independent transmembrane length.

**Table 1:** Secondary structure analyses of KCNE protein sequence to predict transmembrane helices.

	Location of TM within the proteins				GRAVY^5^ coefficient	Estimated TMS (in AA)
	Predict Protein^1^	MENSAT^2^	WaveTM^3^	SAPS^4^		
**KCNE1**	^46^Y-^64^S	^45^A-^66^I	^44^A-^63^L	^44^A-^66^I	1.973 = TM	21
**KCNE2**	^46^F-^70^S	^46^F-^69^V	^46^F-^69^V	^46^F-^72^V	2.288 = TM	25

For the first 4 methods, amino acid positions are given, For the Grand AVerage of hydropathicity (GRAVY) the calculated coefficients are given: a more positive value represents greater hydrophobicity[62].

1 PredictProtein at http://cubic.bioc.columbia.edu/predictprotein.

2 MENSAT at http://saier-144-37.ucsd.edu/memsat.html.

3 WaveTM at http://athina.biol.uoa.gr/bioinformatics/waveTM/.

4 Statistical analysis of protein sequences at http://www.ebi.ac.uk/saps/.

5 GRAVY coefficients of the transmembrane domains of KCNE1 and KCNE2 were calculated using ProtParam at http://ca.expasy.org/tools/protparam.html.

Remarkably, a consistent finding was the presence of a fraction of immature KCNE2 (and to a lesser extent, KCNE1) as well as a slower migrating isoform of KCNE2 predicted to be double the mass from culture media supernatant after ultracentrifugation (Figure 5A). That the higher mass KCNE2 protein collapsed into the lower size with sample heating suggests that it represented a dimer of the 20kDa form (Figure 5B). Moreover, PNGaseF treatment argues against the higher form representing a higher glycosylated species. Whether these proteins represent truly soluble forms of KCNE2, an integral membrane-spanning protein, is unclear at this point (Figure 5C). There is only a single membrane-spanning region in KCNEs. Therefore, it is conceivable that if two KCNE2 subunits were to dimerize by interaction between hydrophobic membrane-spanning regions that the overall aqueous solubility of the oligomer could increase, leading to direct exit from the plasma membrane into the eternal milieu. It is possible that the higher molecular weight species that shifts with boiling represents binding to another protein of equal mass however, this seems less likely given that no other prominent peptide appeared on mass spectrometry analysis.

To summarize, our study shows that despite structural similarity of the KCNE1 and KCNE2 family members that they associate differentially with HERG due primarily to different trafficking rates and characteristics rather than different affinities for the channel. Thus, the constituents of channel complexes in native organs may not simply be a matter of co-expression within a given cell or relative subunit affinity. Protein processing and trafficking may play an important and regulated role in controlling channel characteristics. Questions that these results raise include: do these mechanisms apply to other KCNE-K^+^ channel interactions; are they functional *in vivo*; and are they differentially operational in different tissues?

## Supporting Information

Animation S1 KCNE1-calnexin 3D animation
(9.66 MB AVI)

Animation S2 KCNE2 3D animation
(4.85 MB AVI)
